# Human NAA30 can rescue yeast *mak3∆* mutant growth phenotypes

**DOI:** 10.1042/BSR20202828

**Published:** 2021-03-04

**Authors:** Adrian Drazic, Sylvia Varland

**Affiliations:** 1 Department of Biomedicine, University of Bergen, N-5020 Bergen, Norway; 2 Department of Biological Sciences, University of Bergen, N-5020 Bergen, Norway; 3 Donnelly Centre for Cellular and Biomolecular Research, Toronto, ON M5S 3E1, Canada

**Keywords:** acetyltransferase, MAK3, N-terminal acetylation, NAA30, Saccharomyces cerevisiae, stress response

## Abstract

N-terminal acetylation is an irreversible protein modification that primarily occurs co-translationally, and is catalyzed by a highly conserved family of N-terminal acetyltransferases (NATs). The NatC complex (NAA30–NAA35–NAA38) is a major NAT enzyme, which was first described in yeast and estimated to N-terminally acetylate ∼20% of the proteome. The activity of NatC is crucial for the correct functioning of its substrates, which include translocation to the Golgi apparatus, the inner nuclear membrane as well as proper mitochondrial function. We show in comparative viability and growth assays that yeast cells lacking MAK3/NAA30 grow poorly in non-fermentable carbon sources and other stress conditions. By using two different experimental approaches and two yeast strains, we show that liquid growth assays are the method of choice when analyzing subtle growth defects, keeping loss of information to a minimum. We further demonstrate that human NAA30 can functionally replace yeast MAK3/NAA30. However, this depends on the genetic background of the yeast strain. These findings indicate that the function of MAK3/NAA30 is evolutionarily conserved from yeast to human. Our yeast system provides a powerful approach to study potential human NAA30 variants using a high-throughput liquid growth assay with various stress conditions.

## Introduction

N-terminal acetylation is a common protein modification defined by the addition of an acetyl group to the start of a protein [1–3]. Current estimates suggest that ∼80% of human proteins and ∼50–70% of yeast proteins are to varying degrees N-terminally acetylated [1,4–6]. N-terminal acetylation was discovered more than 60 years ago [7] and its biological significance is becoming increasingly prevalent in many areas of biomedicine. From a clinical perspective, dysregulated N-terminal acetylation can be causative factor of intellectual disability, autism spectrum disorder, and congenital heart disease [8–11]. These clinical manifestations may arise from defects in the developmental programming of organ specifications. A group of enzymes called N-terminal acetyltransferases (NATs) catalyzes N-terminal acetylation, either during or after protein synthesis [12]. At the molecular level, acetylation transforms the positively charged N-terminus into a hydrophobic handle [1,13]. The N-terminal acetylation status influences protein properties such as folding and aggregation [14–19], polymerization [20,21], binding properties [16,22–24] and lifespan [25–27]. By deciding protein fate, the NAT enzymes play essential roles in cell proliferation, apoptosis, protein trafficking, and several other biological processes [12,28].

**Figure 1:**
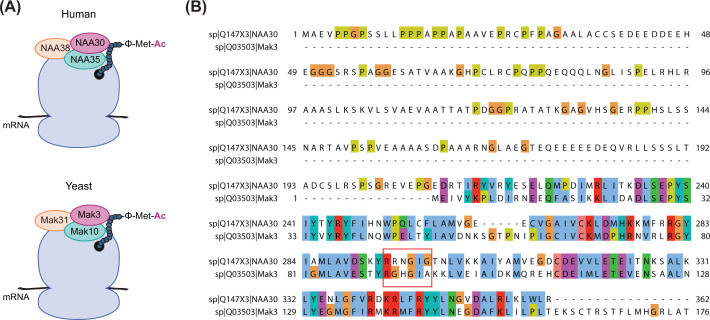
Composition of the NatC complex (**A**) NatC is a trimeric protein complex that consists of the catalytic subunit NAA30 (Mak3), the ribosomal anchor NAA35 (Mak10), and the auxiliary subunit NAA38 (Mak31). The yeast protein names are given in parenthesis. During protein synthesis, NatC can N-terminally acetylate proteins beginning with methionine followed by a hydrophobic or amphipathic amino acid (e.g. for human NAA30 L, I, F, W, V, M and K). Hydrophobic residues are represented by the Φ letter. (**B**) Sequence alignment of human NAA30 and yeast Mak3. The acetyl-CoA-binding domain (RxxGxG/A) is indicated with a red box. Human NAA30 has an N-terminal extension that is not present in the yeast Mak3.

Seven human NAT enzymes have been identified to date (Table 1), where NatA, NatB, and NatC are predominantly responsible for this protein modification. [4,12,29]. NatC is a heterotrimeric complex that contains the catalytic subunit NAA30 (Mak3), the ribosomal anchor NAA35 (Mak10), and the auxiliary subunit NAA38 (Mak31) [3,30,31] (Figure 1A). NatC is conserved from yeast to human with respect to complex formation and enzymatic activity (Figure 1B) [3,31,32]. Human NatC can co-translationally acetylate the N-termini of proteins starting with methionine followed by a hydrophobic or amphipathic residue (Met-Leu/Ile/Phe/Trp/Val/Met/Lys) [33], and several studies suggest that yeast NatC has a similar substrate profile [3,34–36]. The *in vitro* substrate specificity of NatC partly overlaps with NAA50/NatE and NAA60/NatF (see Table 1), but presumably they acetylate distinct substrate classes *in vivo* [6,37,38]. Thus, the presence of a NatC-specific N-terminus does not guarantee that N-terminal acetylation will take place or can predict to which degree a specific NatC-type protein is N-terminally acetylated.

**Table 1:** Overview of yeast and human NATs

NAT enzyme	Catalytic subunit		Auxiliary subunit		Substrate specificity
	Human	Yeast	Human	Yeast	
NatA	NAA10	Ard1	NAA15	Nat1	Ala-, Cys-, Ser-, Thr-, Val-, Gly-
NatB	NAA20	Nat2	NAA25	Mdm20	Met-Asp-, Met-Asn-, Met-Glu-, Met-Gln-
NatC	NAA30	Mak3	NAA35, NAA38	Mak10, Mak31	Met-Leu-, Met-Ile-, Met-Phe-, Met-Trp-, Met-Val-, Met-Met-, Met-His-, Met-Lys-
NatD	NAA40	Nat4			Histones H2A (Ser-Gly-Arg-) and H4 (Ser-Gly-Gly-)
NatE	NAA50	Nat5			Met-Lys-, Met-Val-, Met-Ala-, Met-Tyr-, Met-Phe-, Met-Leu-, Met-Ser-, Met-Thr-
NatF^*^	NAA60	-			Met-Lys-, Met-Ala-, Met-Val-, Met-Met-, Met-Ser-, Met-Leu-, Met-Gln-, Met-Ile-, Met-Tyr-, Met-Thr-, Met-Phe-, Met-Gly-
NatH^*^	NAA80	-			Actins (Asp-Asp-Asp-, Glu-Glu-Glu-)

* Not expressed in yeast.

In the budding yeast *Saccharomyces cerevisiae*, deletion of the individual NatC subunits produces less severe phenotypes compared with NatA or NatB deletion strains [28]. The yeast NatC genes were named according to their ability to maintain and replicate the killer virus M1 (*MAK*; *ma*intenance of *k*iller) [39]. Later studies showed that Mak3 N-terminally acetylates the major viral coat protein *Gag* of the helper virus L-A, and that this modification is necessary for proper viral particle assembly [34,40]. In the absence of particle assembly, the coat protein *Gag* becomes unstable and more susceptible to degradation. Loss of one of the individual NatC subunits also results in reduced growth on non-fermentable carbon sources, such as glycerol and ethanol [3,41,42], indicating that the entire NatC complex is required for proper mitochondrial function. In line with this, depletion of NAA30 disrupts mitochondrial function in human cancer cells and results in reduced levels of mitochondrial matrix proteins, some of which are NatC substrates [33]. Furthermore, overexpression of NAA30 increases cancer cell viability [43]. NatC-related change in organelle function is also observed in the plant *Arabidopsis thaliana*, where N-terminal acetylation by NatC is required for efficient photosynthesis [44]. The mitochondrial and photosynthetic phenotypes could be caused by defective acetylation of one or more mitochondrial and chloroplast protein(s), respectively [40,44]. N-terminal acetylation by yeast NatC is required for protein complex formation (Ubc12–Dcn1) [24,45] as well as subcellular targeting to the Golgi apparatus (Arl3 and Grh1) [46–48], and to the inner nuclear membrane (Trm1-II) [49]. N-terminal acetylation can confer proteins with the ability to interact with other proteins or membranes, which facilitate the subcellular targeting. Golgi targeting of N-terminally acetylated Arl3 is brought about by interaction with Sys1, a Golgi-localized integral membrane protein [46,47].

Given the prevalence and impact of N-terminal acetylation, it will be important to assess the pathogenic nature of newly identified NAT gene variants. We have previously established a yeast-based functional assay to study NAA15 variants [10] and assessed NatA ribosome binding [50]. Anticipating NatC variants, we wanted to explore the possibility for a yeast growth assay to study NAA30 variants. Herein, we report that human NAA30 can rescue several growth defects in yeast *mak3∆* deletion strains. Our yeast growth model represents a simple and powerful approach to rapidly assess the functionality of human NAA30 variants *in vivo*.

## Materials and methods

### Yeast strains

The *S. cerevisiae* haploid strains BY4741 (*MAT****a***; *ura3Δ0; leu2Δ0; his3Δ1; met15Δ0*) and W303-1A (*MAT****a*** *ade2-1 ura3-1 his3-11,15 leu2-3,112 trp1-1 can1-100*) were used in the present study. The *mak3∆* deletion strains (YPR051∆::kanMX6) were constructed by PCR‐mediated gene disruption by replacing the *MAK3* gene with the kanMX6 cassette using homologous recombination. The MAK3-specific KanMX6 module, conferring geneticine resistance in yeast, was PCR amplified from pUC-KanMX6, which was a kind gift from Dr. Zhijian Li, University of Toronto, using the primer pair: MAK3 TEFpr F 5′-CAAGAGATTACAAGATAAAAAAGCCACTACTACAGAAAAGGCGTTGGGTCAGGACacatggaggcccagaataccc-3′ and MAK3 TEFterm R 5′-AATAAAAAAATTGTATTATTAATATATATTTTATCATCATCGAGTGTTTTTCCTTcagtatagcgaccagcattcac-3′. Upper case letters represent sequence with homology to the flanks of *MAK3* while the lower case letters indicate homology to the KanMX cassette. Gene disruption of *MAK3* was confirmed by colony PCR using the primer pair MAK3 F 5′-TGGGTAGGTGCAGTGCTATATT-3′ and MAK3 R 5′-AGAAAGAAGAAGGACCCAAATG-3′, which is positioned 300–450 bp from the start and stop codon of *MAK3*. KanMX6 displacement of *MAK3* generated a larger PCR product, since the kanMX6 module (1357 bp) is bigger than the *MAK3* ORF (531 bp). The WT and *mak3*∆ deletion strains were transformed with the yeast expression vector pBEVY-U (2μ) either empty or containing HA-NAA30 under the *ADH1* promoter or NAA60 under the *GPD* promoter. pBEVY-U-HA-NAA30 [32] and pBEVY-U-NAA60 [6] were previously described, and were a kind gift from Dr. Thomas Arnesen, University of Bergen. Transformants were selected and maintained on SD-Ura medium [0.67% (w/v) yeast nitrogen base without amino acids (BD Difco), 0.2% (w/v) amino-acid supplement powder mixture without uracil [51], 2% (w/v) glucose]. For the NAA60 experiments, the yeast nitrogen base without amino acids and the drop out mix without uracil were from Sigma–Aldrich.

### Yeast protein extraction and immunoblot analysis

Yeast protein extracts were prepared as previously described [52]. In brief, cells in early-log phase were harvested at 5000×***g*** for 10 min at 4°C. The cell pellet was washed twice in cold water and resuspended in 0.1 M NaOH. Following 5 min alkaline treatment at room temperature, the cells were centrifuged at 10000*×**g*** for 1 min and the supernatant was discarded. The cell pellets were resuspended in Laemmli SDS sample buffer (Alfa Aesar), incubated for 5 min at 95°C and centrifuged at 10000*×**g*** for 30 s to remove cell debris; 0.50 mg of wet cell weight was analyzed by immunoblotting. Primary antibodies were rabbit anti-HA-tag (Abcam, ab9110, 1:3000), rabbit anti-NAA30 (Sigma–Aldrich, HPA057824, 1:1000), rabbit anti-NAA60 (Sigma–Aldrich, SAB1102546, 1:1000) and mouse anti-actin (Abcam, ab8224, 1:5000). Secondary antibodies were HRP-conjugated anti-rabbit and anti-mouse (both from GE Healthcare, NA934 and NA931, 1:5000).

### Yeast growth assay on solid media

Yeast cells were grown in SD-Ura medium at 30°C overnight, washed twice in sterile water, and adjusted to 1 OD_600_/ml. Ten-fold serial dilutions in sterile water were spotted (5 μl) on to SD-Ura agar plates supplemented with various chemicals. The plates were incubated at 30°C, if not specified otherwise, for 2–5 days and imaged with a spImager from S&P Robotics. For the chemical treatments, cells were spotted on to plates containing 0.3 M CaCl_2_, 0.1% (w/v) caffeine (5.15 mM), 75 mM hydroxyurea (HU), or 1 M NaCl. To make non-fermentable SD-Ura medium, 2% glucose was replaced with 3% glycerol as the sole carbon source. All experiments were performed three times. Representative results are shown.

### Yeast growth assay in liquid culture

Yeast cells were grown in SD-Ura medium at 30°C overnight, washed twice in sterile water, and adjusted to 1 OD_600_/ml. The cell suspensions (5 μl) were diluted in filtered SD-Ura medium (95 μl) in a 96-well flat-bottom microplate (Greiner, 655180 or Thermo Fisher Scientific, 167008 for the NAA60 experiments), which was subsequently covered with sealing foil (Roche, 04729757001). Yeast cells were incubated at 30°C with continuous shaking and growth was monitored by automatically measuring absorbance at 595 nm at 15-min intervals (6-min for NAA60 experiments) using a Tecan microplate reader. SD-Ura medium with the following carbon sources were used: 2% glucose, 3% glycerol, 2% sodium acetate dihydrate (17 mM), or 2% raffinose, as well as SD-Ura 2% glucose with 1 M NaCl. All experiments were performed in quadruplicates (technical replicates) and at least three times (biological replicates). The growth data within a biological replicate were fitted with logistic non-linear regression growth model and symmetrical (asymptotic) approximate confidence intervals using GraphPad Prism version 8.4.3.686, GraphPad Software, San Diego, California U.S.A., www.graphpad.com. The relative rate constants (k) are given as bar graphs with standard error (SE) indicated. Student’s *t* test was performed to determine the significant difference in growth rates between the wildtype (WT) strain and *mak3∆* with empty vector, *mak3∆* with HA-NAA30 or *mak3∆* with NAA60 using the individual rate constants (k) within a biological experiment (*P≤0.05; **P≤0.01; ***P≤0.00; *n*≥3).

## Results

### Human NAA30 stably expresses in yeast

Structural studies have provided important information on how the NAT enzymes recognize their substrates [53]. The heterotrimeric NatC complex is the only human NAT enzyme for which a detailed structure or functional analysis has not yet been reported. Yeast Mak3 and human NAA30 share limited sequence identity (19.8%), but both have the recognizable Gcn5-related N-acetyltransferase (GNAT) fold (Figure 1B). However, previous work has shown that in the case of the NAT enzyme family, sequence similarity does not necessarily correlate with structural and functional similarity [54]. Human NAA30 has a N-terminal extension that is neither present among the other catalytic human NAT subunits, nor in the yeast homolog Mak3. The function of the N-terminal segment remains unknown. A recent crystal structure revealed the molecular interactions between the three NatC subunits in budding yeast [36]. Structural comparisons with the heterodimeric NatA and NatB complexes unraveled some distinguished differences and established the modus operandi of the NatC complex [36]. We have previously used yeast growth assays to study pathogenic variants of NAA15, which encode the ribosome binding subunit of the NatA complex [10]. To study the impact of possible NatC variants *in vivo*, we have explored the consequences of deleting *MAK3*, the catalytic subunit of the NatC complex and the yeast homolog of human NAA30.

Loss of the individual NatC subunits results in slower growth on non-fermentable medium [3,41,42]. To further explore the functional effects of *MAK3* deletion, we performed growth experiments at different stress conditions. We wanted to determine the importance of *MAK3* for yeast viability in response to various environmental stressors as well as to compare the strength of the assays by using different yeast strains and methods. To this end, the *MAK3* gene was deleted in the two widely used yeast strains BY4741 and W303-1A. BY4741 is derived from S288c and was used in the *S. cerevisiae* genome deletion project. W303-1A is a commonly used model organism in aging research and was generated by crossing a series of yeast strains of uncertain genealogy [55]. Genetic analysis revealed that the W303 genome has a similarity of 85.4% compared with S288c, with the remaining genetic differences affecting 799 proteins [55]. The genetic variations between BY4741 and W303-1A contribute to some phenotypic differences. W303-1A cells have increased cell size and volume compared with BY4741 cells. Furthermore, W303-1A cells display increased tolerance to alkali-metal ions as well as increased sensitivity to oxidative stress [55,56]. Because of their diverse genetic backgrounds, stressors affect BY4741 and W303-1A cells differently. Both strains have previously been used to study the physiological role of NatA [5,50,57] and NatC [32,58], respectively. Since not all NatC substrates are known, we used the two different yeast strains to determine if one of them was better suited to study MAK3/NAA30.

**Figure 2:**
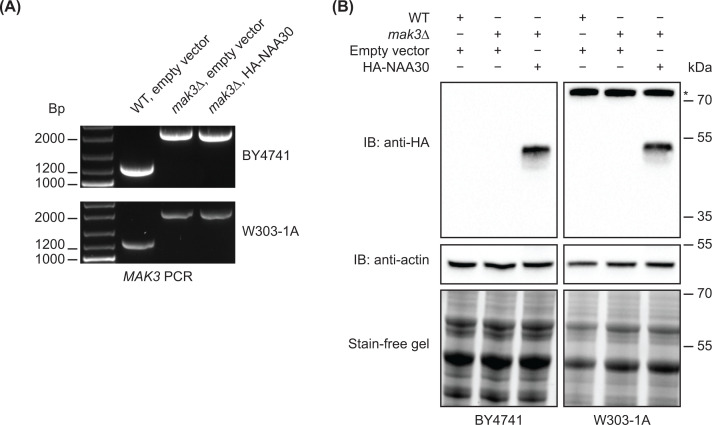
Confirming *MAK3* deletion and HA-NAA30 expression (**A**) Deletion of *MAK3* was achieved by homologous recombination by replacing the *MAK3* ORF with a KanMX cassette conferring geneticine resistance in yeast. Gene disruption of *MAK3* in the *mak3∆* deletion strains was confirmed by colony PCR using a primer pair that was positioned 300–450 bp from the start and stop codon of *MAK3*. *MAK3* PCR product 1303 bp (*MAK3* 531 bp + 772 bp flanking region) and *MAK3::kanMX6* PCR product 2128 bp (KanMX6 1356 bp + 772 bp flanking region). (**B**) Expression of HA-NAA30 was confirmed by immunoblotting using anti-HA. Actin and stain-free imaging served as loading controls. Asterisk (*) indicates unspecific band in the W303-1A strain background. Full immunoblot images shown in Supplementary Figure S1.

The *mak3∆* strains were constructed by PCR-based gene disruption, by replacing the *MAK3* gene with a kanMX6 cassette, conferring geneticin resistance in yeast, using homologous recombination. The kanMX6 cassette was PCR amplified using primers that contained 55 bp homologous to the flanks of the *MAK3* ORF. The replacement of *MAK3* with KanMX6 was confirmed by colony PCR, as the KanMX6 cassette (1357 bp) is larger than *MAK3* (531 bp) (Figure 2A). We then transformed the resulting *mak3∆* deletion strains with a high copy plasmid (pBEVY-Ura, 2μ) expressing HA-NAA30 under the *ADH1* promoter. Immunoblotting confirmed similar protein expression levels of HA-NAA30 in both yeast strain backgrounds (Figure 2B and Supplementary Figure S1).

### Human NAA30 rescues yeast *mak3∆* growth defects

**Figure 3:**
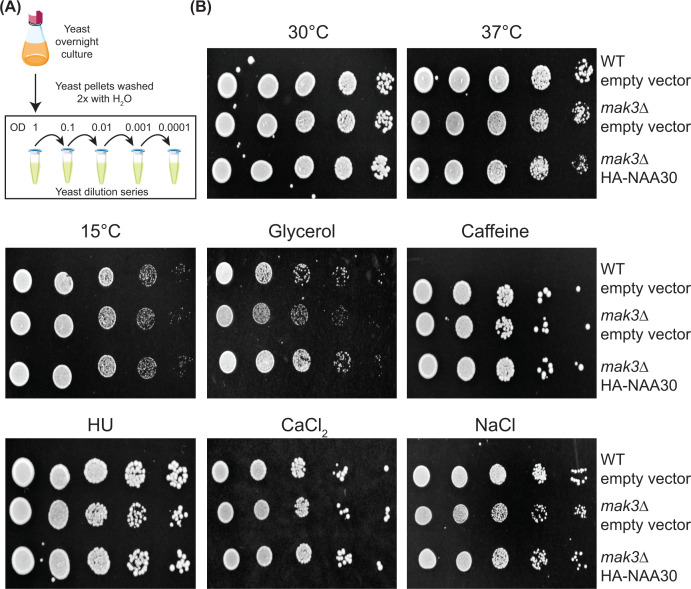
Deletion of *MAK3* impairs growth on glycerol and sodium chloride (**A**) Schematic of the experimental setup. BY4741 WT and *mak3∆* mutant strains transformed with an empty vector or plasmid expressing HA-NAA30 were ten-fold serial diluted, spotted on to SD-Ura plates with various stressors and incubated at 30°C, if not indicated otherwise. (**B**) The plates were incubated for 2 days for 30°C, 3 days for 300 mM CaCl_2_, 0.1% (w/v) caffeine (5.15 mM) and 75 mM HU, 4 days for 37°C and 1 M NaCl, and 5 days for 15°C and 2% glycerol. All experiments were performed three times. Representative results are shown.

Next, we wanted to assess whether *MAK3* supports yeast growth in various conditions, including different temperatures, carbon sources, and chemicals targeting different cellular components and processes. First, we performed growth experiments with the BY4741 strain using a classical spot assay (Figure 3A). To do so, we prepared SD-Ura plates with 2% glucose supplemented with different chemicals, such as 0.3 M CaCl_2_, 0.1% (w/v) caffeine, 75 mM HU, 1 M NaCl or substituted glucose with 3% glycerol as the sole carbon source (Figure 3B). Consistent with previous studies, deletion of *MAK3* did not cause a detectable growth defect within the resolution of the spot assay on SD-Ura with 2% glucose at 30°C, confirming that *MAK3* is not essential for yeast viability under normal growth conditions. The addition of stress factors such as cold stress (15°C), heat stress (37°C), caffeine, HU, and CaCl_2_ did not cause any noticeable growth effects either. However, the *mak3∆* mutant cells displayed a minor growth defect during salt stress using 1 M NaCl and grown on glycerol as sole carbon source. Overexpression of human NAA30 in the *mak3∆* deletion strain rescued these subtle growth defects. This finding demonstrates that human NAA30, although similar in the catalytic GNAT fold, but differing substantially in the N-terminal region (Figure 1B), is capable of complementing yeast Mak3. In the yeast Arl3 localization model, complementation of Mak3 requires the presence of the ribosomal binding subunit Mak10/NAA35 [32], suggesting that N-terminal acetylation still takes place co-translationally due to the formation of an interspecies hybrid NatC complex. Moreover, it suggests that the N-terminal extension of NAA30 has more of a supportive effect. The co-evolution of the NatC subunits enabled us to study the individual subunits without humanizing the entire complex, which is the case for NatA and NatB [5,59]. From the rather subtle growth defects that we observed using the spot assay, it became clear that Mak3 is not as crucial as Ard1/NAA10 or Nat1/NAA15 of the NatA complex for cell viability. The NatA complex appears essential in human cells while yeast NatA mutants are viable, however showing a diminished growth and a higher sensitivity to various stressors [5]. These strong growth phenotypes have allowed us to use growth on solid media as a proxy for NatA function *in vivo* [10,50]. Because the spot assays only revealed subtle growth defects upon *MAK3* deletion, we decided to shift to growth experiments in liquid culture.

### Subtle *mak3∆* growth defects are captured by high-throughput liquid growth assays

**Figure 4:**
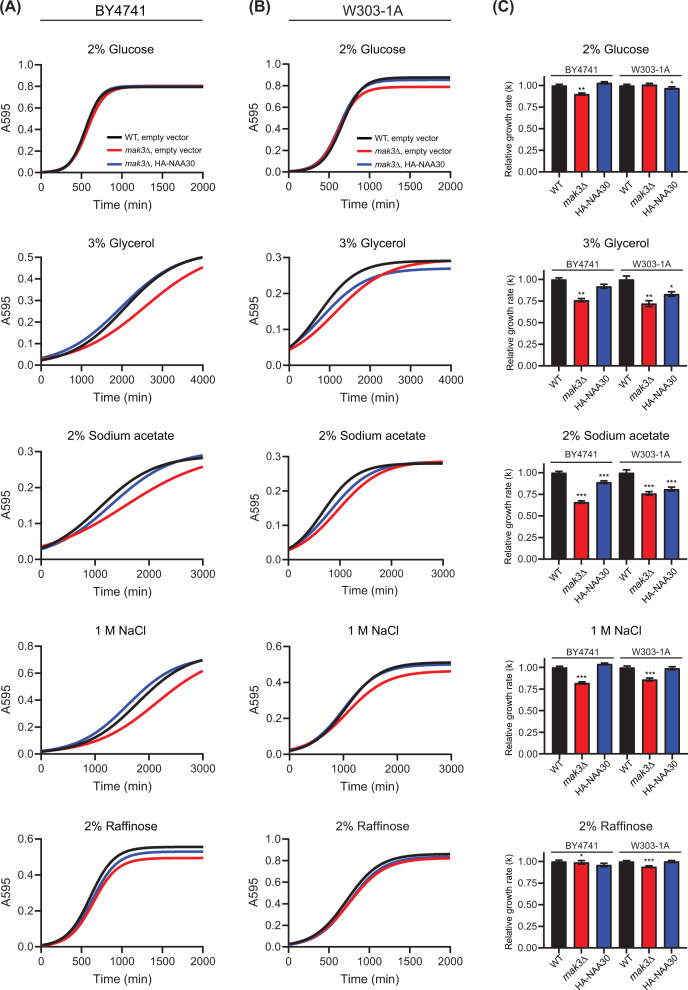
High-throughput assay to monitor growth of *mak3∆* mutant strain in response to various stressors BY4741 (**A**) and W303-1A (**B**) WT and *mak3∆* mutant cells transformed with empty pBEVY-U vector or HA-NAA30 plasmid were grown in SD-Ura medium in a 96-well culture plate at 30°C. Yeast growth was monitored by measuring absorbance at 595 nm every 15 min using a microplate reader. (**C**) The growth data were fitted with logistic non-linear regression growth model using GraphPad Prism 8. The relative growth rates (k) are shown as bar graphs with SE indicated. All experiments were performed in quadruplicates (technical replicates) and at least three times (biological replicates). Representative results are shown. Student’s *t*-test was performed to determine the significance of growth rates between the WT strain and *mak3∆* with empty vector or *mak3∆* with HA-NAA30 using the individual rate constants (k) within a biological experiment (*P≤0.05; **P≤0.01; ***P≤0.00; *n*≥3).

Subtle growth effects are difficult to capture by spot assays and many (non-)effects could be misinterpreted or even be disregarded by solely relying on this method as a readout. Furthermore, the preparation of the plates including addition of chemical compounds is sometimes challenging to handle regarding reproducibility. Therefore, we decided to investigate the growth of *mak3∆* mutant strains during different stress conditions using a liquid growth assay where the yeast strains were cultivated in a 96-well format and the growth was monitored using an absorbance microplate reader (Figure 4). We prepared SD-Ura medium containing 2% glucose, 3% glycerol, 2% sodium acetate, or 2% raffinose, as well as SD-Ura 2% glucose with 1 M NaCl. Surprisingly, in contrast with the experiments on solid media, the BY4741 *mak3∆* deletion strain displayed a subtle growth defect at 30°C in 2% glucose (Figure 4A,C; Table 2). Although the decreased growth rate was not dramatic, it was reproducible. The *mak3∆* mutant strain also displayed a slightly reduced growth rate in raffinose, another fermentable carbon source. However, the most noticeable difference was the inability of *mak3∆* cells to reach the same growth maximum as WT cells in the raffinose medium (Table 2). Interestingly, deletion of *MAK3* significantly reduced the cells ability to grow in non-fermentable conditions (2% glycerol and 2% sodium acetate) and during salt stress (1 M NaCl). In agreement with our spot assays, overexpression of human NAA30 rescued the growth defects during these conditions, although the exponential growth rates were not completely restored in glycerol or sodium acetate containing medium.

Next, we wanted to investigate if these findings were reproducible in another yeast background, namely W303-1A (Figure 4B,C; Table 2). We observed comparable results for the W303-1A strain as in the BY4741 strain. In this case, the *mak3∆* cells did not display a significant change in the exponential growth rate in glucose containing media, but maximum growth was decreased compared with WT cells. Furthermore, the *mak3∆* cells showed diminished growth in glycerol, sodium acetate, and NaCl medium. Interestingly, we were not able to rescue all growth defects to the same extent by expressing human NAA30 as in the BY4741 strain background. This was especially noticeable for the non-fermentable conditions, glycerol, and sodium acetate. In the W303-1A background, overexpression of human NAA30 caused a partial rescue of the exponential growth rate in glycerol, but the cells did not reach the same growth maximum as the WT cells. In sodium acetate, however, the *mak3∆* mutant cells expressing human NAA30 reached the same plateau phase as WT cells, but the exponential growth rate was only partially rescued. Sodium acetate stress response involves two components, namely sodium and acetate ions. Acetate ions may inhibit cell growth by perturbing cytosolic pH homeostasis. Sodium ions inhibits growth in two ways: it creates a hyperosmotic environment and increases intracellular sodium levels which concomitantly decreases intracellular potassium levels [60]. Our results indicate that this ‘double stress’ caused by sodium acetate potentiates the effects caused by NaCl, and results in a growth defect that cannot be fully rescued by overexpression of human NAA30. In summary, we show that *mak3∆* mutant cells display decreased fitness in medium containing the non-fermentable carbon sources glycerol and sodium acetate and have decreased resistance to salt stress.

**Table 2:** Logistic growth model parameters for the experimental data

Logistic growth	BY4741			W303		
Best-fit values	WT, empty vector	*mak3∆*, empty vector	*mak3∆*, HA-NAA30	WT, empty vector	*mak3∆*, empty vector	*mak3∆*, HA-NAA30
**2% Glucose**						
YM	0.7947	0.7983	0.8035	0.8765	0.7887	0.8537
Y0	0.0026	0.0036	0.0021	0.0040	0.0049	0.0059
k (× 10^−3^)	10.5 ± 0.13	9.42 ± 0.11	10.8 ± 0.14	8.13 ± 0.11	8.19 ± 0.12	7.92 ± 0.10
**3% Glycerol**						
YM	0.5265	0.5411	0.5307	0.2908	0.2942	0.2699
Y0	0.0224	0.0267	0.0323	0.0492	0.0430	0.0509
k (× 10^−3^)	1.51 ± 0.03	1.15 ± 0.02	1.39 ± 0.03	2.14 ± 0.08	1.55 ± 0.05	1.79 ± 0.05
**2% Sodium acetate**						
YM	0.2892	0.2981	0.3046	0.2803	0.2876	0.286
Y0	0.0309	0.0355	0.0284	0.0312	0.0276	0.0325
k (× 10^−3^)	1.95 ± 0.03	1.29 ± 0.02	1.75 ± 0.03	3.08 ± 0.10	2.34 ± 0.05	2.50 ± 0.05
**2% Raffinose**						
YM	0.5559	0.4944	0.5300	0.8624	0.8245	0.8368
Y0	0.0088	0.0065	0.0082	0.0252	0.0254	0.0207
k (× 10^−3^)	6.78 ± 0.10	6.69 ± 0.13	6.51 ± 0.12	4.87 ± 0.05	4.57 ± 0.04	4.87 ± 0.05
**1 M NaCl**						
YM	0.7532	0.7598	0.7296	0.5120	0.4643	0.5005
Y0	0.0177	0.0191	0.0220	0.0192	0.0247	0.0215
k (× 10^−3^)	2.08 ± 0.03	1.71 ± 0.02	2.16 ± 0.02	3.13± 0.05	2.69 ± 0.04	3.10 ± 0.05

YM is the maximum population, Y0 is the starting population, k is the rate constant with SE.

An interesting observation was that the degree of rescue of *mak3* deletion mutants by human NAA30 varied between BY4741 and W303-1A. The two yeast strains have different genetic backgrounds leading to different physiological properties, one being tolerance to salt stress [56]. Because the yeast NatC acetylome has not been systematically mapped, relatively few NatC substrates are known. Trying to explain possible reasons for the observed difference in NAA30 rescue is thus not trivial. Because NatC deletion strains display reduced growth on non-fermentable carbon sources, such as glycerol and sodium acetate, we hypothesize that the difference in rescue could be related to mitochondrial function and stress resistance mechanisms. This is in line with the notion that human NAA30 has a role in maintaining mitochondrial integrity [33]. It is worth noting that BY4741 and W303-1A strains have different respiratory capacities [61], owing partly to BY4741 carrying a mutant form of the *HAP1* gene, which encodes a transcription factor that functions during aerobic growth [62].

### Human NAA60 rescues *mak3∆* growth defect in glycerol

**Figure 5:**
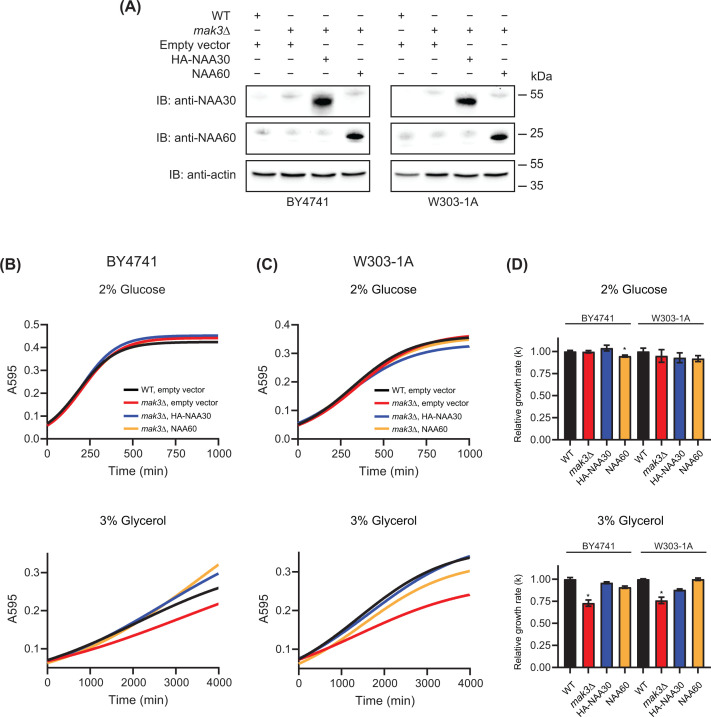
Human NAA60 rescues growth defect of *mak3∆* cells (**A**) Expression of HA-NAA30 and NAA60 was confirmed by immunoblotting using anti-NAA30 and anti-NAA60. Anti-actin served as loading control. Full immunoblot images shown in Supplementary Figure S2. BY4741 (**B**) and W303-1A (**C**) WT and *mak3∆* mutant cells transformed with empty pBEVY-U vector, HA-NAA30 or NAA60 plasmid were grown in SD-Ura medium in a 96-well culture plate at 30°C. Yeast growth was monitored by measuring absorbance at 595 nm every 6 min using a microplate reader. (**D**) The growth data were fitted with logistic non-linear regression growth model using GraphPad Prism 8. The relative growth rates (k) are shown as bar graphs with SE indicated. All experiments were performed at least in triplicates (technical replicates) and three times (biological replicates). Representative results are shown. Student’s *t*-test was performed to determine the significance of growth rates between the WT strain, *mak3∆* with empty vector, *mak3∆* with HA-NAA30, *mak3∆* with NAA60 or using the individual rate constants (k) within a biological experiment (*P≤0.05; *n*≥3).

Human NAA30 and NAA60 have overlapping substrate specificities (see Table 1) [6,33] and it was previously shown that NAA60 is able to rescue the Golgi localization of Arl3-GFP in *mak3∆* mutant cells [32]. To validate our experimental approach, we next investigated whether overexpression of human *NAA60* could rescue the growth phenotype of *mak3Δ* mutant strains on glycerol (Figure 5A and Supplementary Figure S2). The ectopic expression of *NAA60* did not considerably alter the growth rates of *mak3∆* mutants in glucose medium (Figure 5B–D). Interestingly, NAA60, which in human cells is associated with the Golgi [38], partly rescued the *mak3Δ* glycerol growth phenotype in the BY4741 background, and even fully rescued this growth defect in W303-1A (Figure 5C,D).

Growth analysis of *mak3∆* mutant cells grown in SD-Ura supplemented with 3% glycerol showed that the expression of human *NAA30* or *NAA60* resulted in relative growth rates (k) that closely resembled the WT strains (Figure 5D). However, we noticed that *mak3∆* mutants expressing *NAA60*, in both strain backgrounds, exhibited a longer lag phase compared mutant cells expressing *NAA30* (Supplementary Figure S3). This was followed by an accelerated exponential phase. The *mak3Δ* strains rescued by NAA30 displayed growth curves that were nearly identical with the WT strains, indicating a more direct effect on the yeast cells. One possible explanation for this observation is that NAA30 and NAA60 use different enzymatic mechanisms to acetylate NatC substrate(s) that are essential for glycerol growth. A previous study demonstrated that human NAA60 most likely acts freely in the yeast cytosol and is not associated with the Golgi membrane as in human cells [32]. One could hypothesize that NAA60 post-translationally acetylates NatC substrates, whereas NAA30 acts co-translationally due to direct interaction with the yeast NatC complex. This can lead to a slower adaptation of the NAA60-expressing yeast cells, which consequently results in a delayed rescue effect.

### MAK3 supports growth in both rich and synthetic glycerol medium

**Figure 6:**
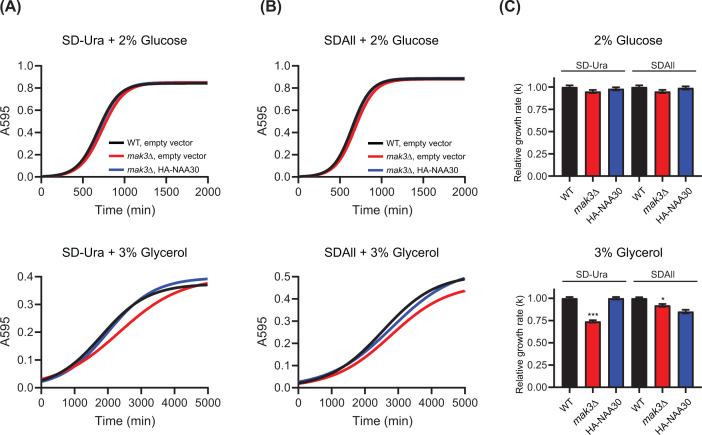
Human NAA30 rescues growth defect of *mak3∆* cells in glycerol medium WT (BY4741) and *mak3Δ* mutant cells transformed with empty pBEVY-U vector or HA-NAA30 plasmid were grown overnight in SD-Ura medium. Cells were washed and resuspended in sterile water. Liquid growth assays were performed by culturing the yeast cells in 96-well plate at 30°C in (**A**) SD-Ura or (**B**) SDAll medium with 2% glucose or 3% glycerol as sole carbon source. Yeast growth was monitored by measuring absorbance at 595 nm every 15 min using a microplate reader. (**C**) Growth data were fitted with logistic non-linear regression growth model. The relative growth rates (k) are shown as bar graphs with SE indicated. Student’s *t*-test was performed to determine the significant differences in growth rates between the WT strain and *mak3∆* strains using the individual rate constants (k) (*P≤0.05; ***P≤0.001; *n*=3).

The first studies showing that *mak3∆* mutants have a respiratory defect used rich YPG medium at 30°C [41] or 37°C [3]. In this study, we used a synthetic defined medium (SD-Ura) to select for yeast cells having the pBEVY-Ura expression plasmid, which may partly mask growth defects by slowing growth in itself. To exclude that the lack of uracil had any major effect, we performed the same growth assay with the BY4741 strain using both SD-Ura (selection) and SDAll medium (no selection) (Figure 6, Table 3). As expected, the cells showed a higher growth maximum in SDAll medium than in the SD-Ura medium, regardless of whether the carbon source was glucose or glycerol. Furthermore, the *mak3∆* mutant cells showed a reduced respiratory growth on glycerol compared with the fermentative growth on glucose. Overexpression of human NAA30 rescued the growth defects in both glycerol media, and the exponential growth rate was almost completely recovered in the SD-Ura medium. That is, the *mak3Δ* mutant cells were able to retain the HA-NAA30 plasmid even in the absence of selection. The reduced growth of *mak3Δ* mutant cells in glycerol medium most likely provides a strong selective pressure on cell viability, causing the cells to keep the HA-NAA30 plasmid.

**Table 3:** Logistic growth model parameters for BY4741 strains grown in SD-Ura or SDAll medium

Logistic growth	WT, empty vector	*mak3∆*, empty vector	*mak3∆*, HA-NAA30	WT, empty vector	*mak3∆*, empty vector	*mak3∆*, HA-NAA30
**Best-fit values**	**SD-Ura + 2% glucose**			**SDAll + 2% glucose**		
YM	0.8423	0.8488	0.8509	0.8829	0.8795	0.888
Y0	0.0037	0.0034	0.0037	0.0029	0.0027	0.0028
k (× 10^−3^)	7.95 ± 0.15	7.55 ± 0.13	7.80 ± 0.14	8.89 ± 0.17	8.48 ± 0.15	8.82 ± 0.16
	**SD-Ura +3 % glycerol**			**SDAll + 3% glycerol**		
YM	0.3742	0.3983	0.3973	0.5108	0.4699	0.542
Y0	0.0261	0.0306	0.0219	0.0206	0.0185	0.026
k (× 10^−3^)	1.43 ± 0.02	1.07 ± 0.01	1.43 ± 0.02	1.25 ± 0.01	1.15 ± 0.02	1.06 ± 0.02

YM is the maximum population, Y0 is the starting population, k is the rate constant with SE.

## Discussion

The NatC complex is conserved from yeast to humans with respect to subunit composition, ribosome binding, and substrate specificity (Figure 1A) [3,31,33]. Early studies suggested that the complete NatC complex was required for N-terminal acetylation of NatC-type substrates *in vivo*, since loss of the individual NatC subunits resulted in the same abnormal phenotypes, namely reduced growth on non-fermentable carbon sources and inability to propagate the L-A virus [3,34,40–42]. Later studies showed that NatC-mediated N-terminal acetylation was required for correct targeting of Arl3 to the Golgi [46,47]. In yeast cells lacking *MAK3* or *MAK10* Arl3-GFP was diffusely distributed through the cytoplasm, instead of displaying its normal punctuate pattern. The diffuse Arl3 localization phenotype was, however, not observed in cells lacking *MAK31* [47]. Mass spectrometry further confirmed that Arl3 was N-terminally acetylated in the *mak31Δ* deletion strain [47]. Thus, Mak31 is not required for all NatC-mediated acetylation reactions. Furthermore, a *mak31Δ ypt6Δ* double mutant is synthetically lethal [63], suggesting that Mak31 may be required for a protein that is essential in the *ypt6Δ* mutant background. In terms of *in vivo* protein function, human NAA30 can functionally replace yeast Mak3 in certain cases without humanizing the other NatC subunits. The Arl3 localization phenotype was later used as a model system to investigate functional conservation and redundancy of NATs [32]. NAA30 was able to restore Golgi localization of Arl3, but the rescue required the presence of either yeast Mak10 or human NAA35. This result suggests that NAA30 interacts with Mak10 and forms a human-yeast interspecies NatC complex. This contrasts with the rescue of NatA or NatB growth phenotypes [5,59], where both the catalytic and ribosomal human subunits are required to complement the respective yeast NAT enzyme. Interestingly, *mak3Δ ric1Δ* double mutants are also synthetic lethal, but this effect can be suppressed by human NAA30 [46]. Moreover, NAA30 from *A. thaliana* (AtMAK3) can functionally replace yeast NatC [44]. AtMak3 expression in a *mak3∆ mak10∆* double mutant yeast strain restored growth in non-fermentable glycerol medium, as well as the ability to propagate the L-A virus.

In the present study, we show that human NAA30 can rescue the *mak3∆* growth defect on non-fermentable carbon sources, both on solid agar and in liquid culture. These findings correlate with human NAA30 being able to restore the Golgi localization of Arl3 [32,47]. However, we do not know whether the rescue of the glycerol phenotype requires the presence of Mak10/NAA35 as is the case for Arl3. Further studies are required to fully understand NatC subunit interdependence and the functional role of Mak31/NAA38. Although, the human NatC structure has not been solved, yet, the structure of budding yeast NatC was recently presented [36]. It demonstrates how Mak3/NAA30 and Mak10/NAA35 interact for substrate recognition as well as ribosome binding. Nevertheless, the distinct role for Mak31/NAA38 in the NatC complex could not be sufficiently clarified [36].

Here, we used an N-terminally HA-tagged version of NAA30, which does not interfere with NatC activity. The present study shows that NAA30 is important for cellular respiration and response to salt stress. This is not the first time that the NatC complex has been linked to modulating stress resistance. In the worm *Caenorhabditis elegans* loss-of-function mutation in natc-1/NAA35 results in increased tolerance to transition metals, heat stress, and oxidative stress, suggesting that a functional NatC complex is crucial for normal sensitivity to a wide range of stressors [64,65].

By using two separate methods, we demonstrated that human NAA30 shows N-terminal acetylation activity towards some endogenous yeast NatC substrates. In our hands, the spot assay showed less sensitivity compared with the liquid growth assay. The spot assay is suitable for strains with severe growth defects, which is the case for NatA mutants [5,10,50]. The liquid growth assay draws a more refined picture for the subtle growth defects that we observed in the present study. Further advantages of monitoring growth using a microplate reader include automated data collection and a time saving high-throughput format, allowing a high number of parallel conditions and replicates. Another aspect to consider is that we used two different yeast strains; BY4741 and W303-1A, which have different genetic backgrounds [55]. Although, both BY4741 and W303-1A showed similar results regarding growth rates and defects upon *mak3∆* deletion, there were also distinct differences, especially regarding the rescue abilities of human NAA30 and NAA60, which shares overlapping substrate specificities with the NatC complex [6,33]. Therefore, the obtained results in such viability assays must be carefully assessed, especially in respect to stress factors, experimental method, and genetic background of the model organism. Nevertheless, our data also demonstrate that the NatC complex acts on various substrates, whose N-terminal acetylation status display distinguished effects on the yeast cells, depending on their genetic background, as well as their stress response.

The complexity and numbers of NAT enzymes increases in higher evolved eukaryotic organisms, which can perform both co- and post-translational N-terminal acetylation [4,12]. Whereas yeast only expresses the five NAT enzymes, NatA to NatE, humans express at least seven identified NATs (NatA to NatF and NatH) and more NAT enzymes are likely to be discovered. Yeast can serve as a valuable model system for investigating mammalian NAT enzymes. By using a yeast strain lacking NatB activity (*nat3/naa20∆*), we have previously showed that the highly specialized actin NAT NAA80/NatH [20,66,67] holds the intrinsic capacity to post-translationally acetylate yeast NatB-type substrates *in vivo*, including actin (Act1) [68]. Considering that all NatC subunits are required for proper cellular respiration, the liquid growth assay we have developed, based on reduced growth in glycerol and sodium acetate, can easily be expanded to investigate the functionality of human NAA30 variants *in vivo*, including NAA35 and NAA38 variants.

## Supplementary Material

Supplementary Figures S1-S3

## Data Availability

Data and materials are available upon request.

## Abbreviations

GNAT: Gcn5-related N-acetyltransferase
HU: hydroxyurea
MAK: MAintenance of Killer
NAA: Nα-terminal acetyltransferase
NAT: N-terminal acetyltransferase
WT: wildtype
