# Supplementary material for: Human NAA30 can rescue yeast mak3∆ mutant growth phenotypes
Source: Biosci Rep. 2021 Mar 4;41(3):BSR20202828. doi: 10.1042/BSR20202828 (PMC7938456; doi:10.1042/BSR20202828)
Supplement: Supplementary Figures S1-S3 [file BSR-2020-2828_supp.pdf]

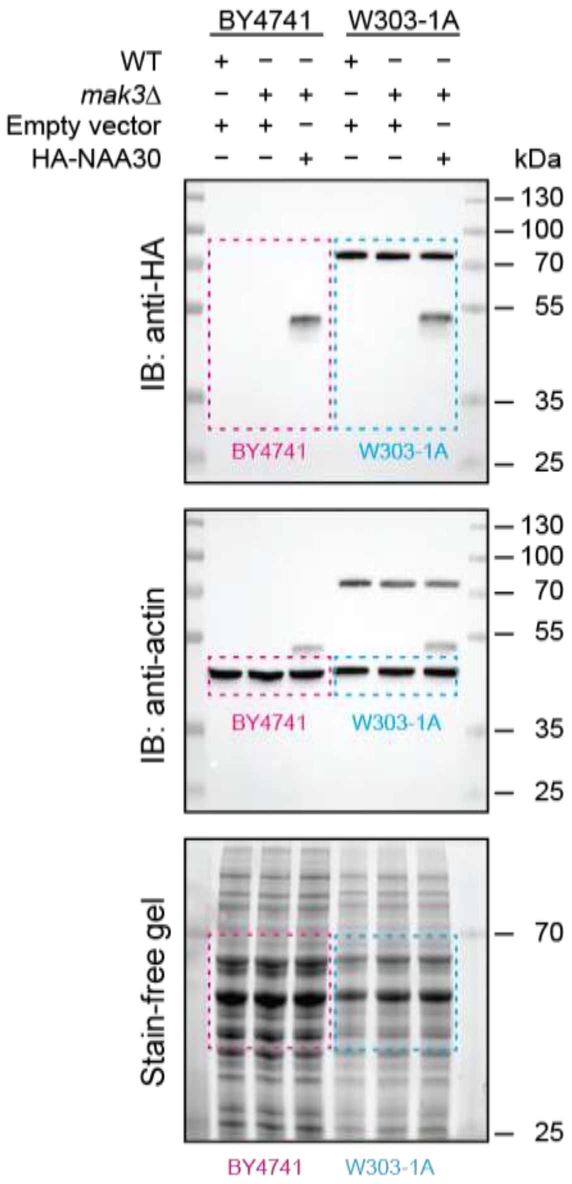

Supplementary Fig. 1. Uncropped immunoblot images for Fig 2. panel B.

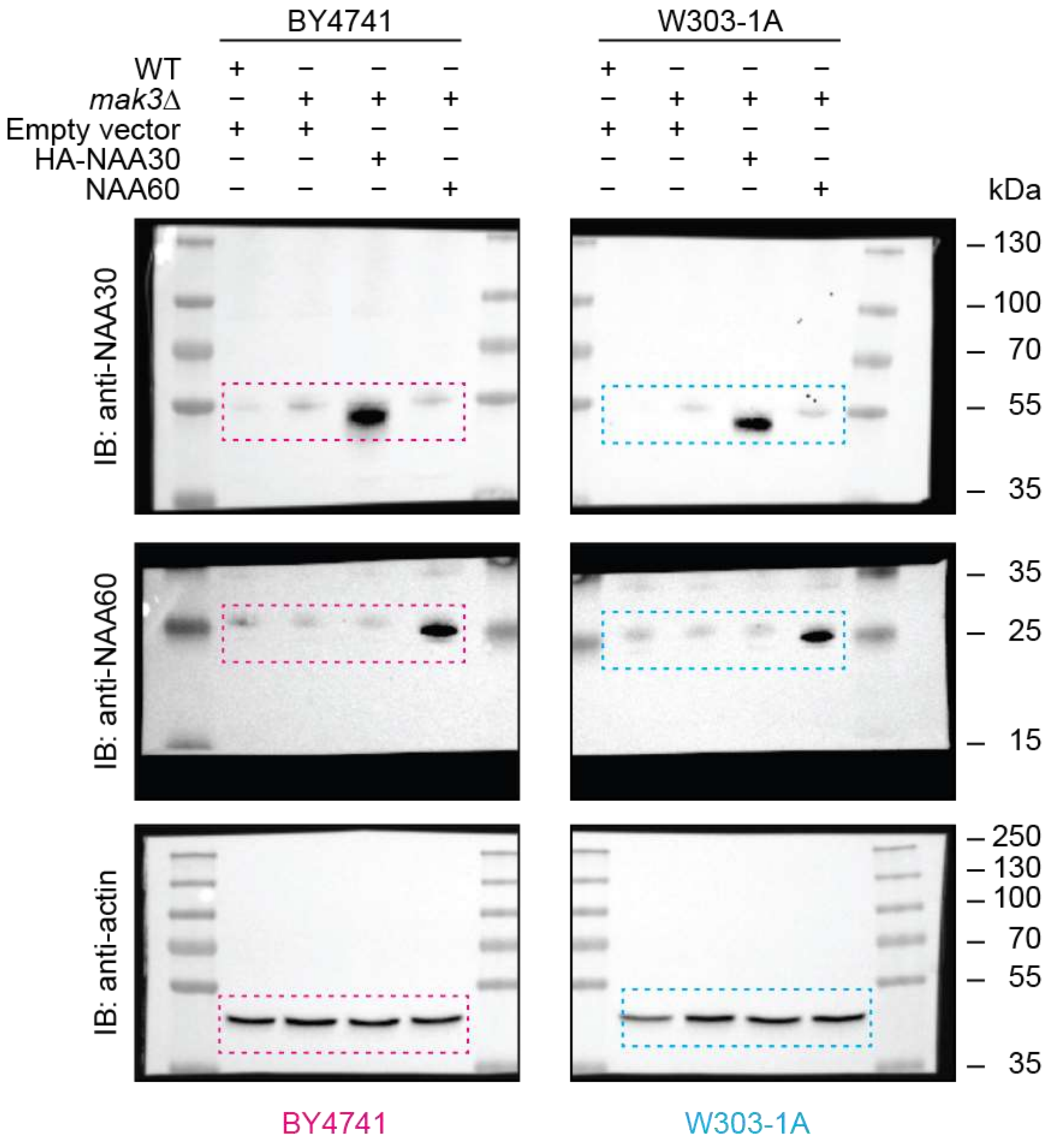

Supplementary Fig. 2. Uncropped immunoblot images for Fig 5. panel A.

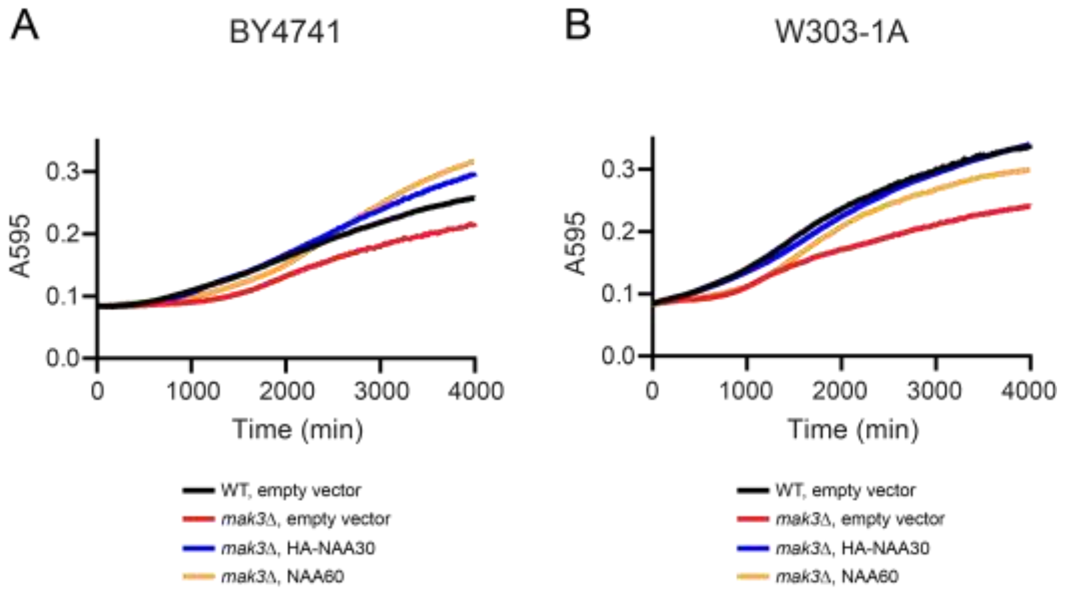

Supplementary Fig. 3. *mak3*Δ mutant strains ectopically expressing human NAA60 show prolonged lag phase in glycerol medium. Growth curves corresponding to fitted data in Fig 5, panel B-D. BY4741 (A) and W303-1A (B) wild type and *mak3*Δ mutant cells were transformed with empty pBEVY-U vector, HA-NAA30 or NAA60 plasmid. Cells were grown in SD-Ura medium supplemented with 3% glycerol in a 96-well plate at 30°C. Yeast growth was monitored by measuring absorbance at 595 nmevery 6 minutes using a microplate reader. Shown is average growth curve of four technical replicates.
